# Ecological Momentary Assessment and mHealth Interventions Among Men Who Have Sex With Men: Scoping Review

**DOI:** 10.2196/27751

**Published:** 2021-08-03

**Authors:** Viktor Clark, Sunny Jung Kim

**Affiliations:** 1 Department of Health Behavior and Policy School of Medicine Virginia Commonwealth University Richmond, VA United States

**Keywords:** mHealth, men who have sex with men, mobile health, interventions, mental health, sexual health, ecological momentary assessment, behavior

## Abstract

**Background:**

Ecological momentary assessment (EMA) is a research design that allows for the measurement of nearly instantaneous experiences within the participant’s natural environment. Using EMA can help improve recall bias, ecological validity, and patient engagement while enhancing personalization and the ubiquity of interventions. People that can benefit from the use of EMA are men who have sex with men (MSM). Previous EMA studies have been successful in capturing patterns of depression, anxiety, substance use, and risky sexual behavior. These findings are directly relevant to MSM, who have high rates of each of these psychological and behavioral outcomes. Although there is a driving force behind the growing literature surrounding EMAs among MSM, no synthesizing reviews yet exist.

**Objective:**

The aims of this study were to (1) synthesize the literature across fields on how EMA methods have been used among MSM, (2) better understand the feasibility and acceptability of EMA interventions among MSM, and (3) inform designs for future research studies on best evidence-based practices for EMA interventions.

**Methods:**

Based on 4 library databases, we conducted a scoping review of EMAs used within interventions among MSM. The eligibility criteria included peer-reviewed studies conducted in the United States and the use of EMA methodology in an intervention for MSM. Modeling after the Centers for Disease Control and Prevention’s Compendium of Evidence-Based Interventions as the framework, we applied a typology that used 8 distinct review criteria, for example, sample size, design of the intervention, random assignment, design of the follow-up investigation, rate of retention, and rate of engagement.

**Results:**

Our results (k=15, N=952) indicated a range of sample sizes; the smallest sample size was 12, while the largest sample size was 120. Of the 15 studies, 7 (47%) focused on outcomes related to substance use or outcomes related to psychological experiences. Of the 15 studies, 5 (33%) implemented an EMA intervention across 30 days. Of the 15 studies, 2 studies (13%) used random assignment, and 2 studies (13%) had quasi-experimental designs. Of the 15 studies, 10 studies (67%) reported acceptable retention rates greater than 70%. The outcomes that had event-contingent prompts (ie, prompts after engaging in substance use) were not as effective in engaging participants, with overall engagement rates as low as 37%.

**Conclusions:**

Our systematic scoping review indicates strong evidence that the EMA methodology is both feasible and acceptable at high rates among MSM, especially, when examining psychological and behavioral outcomes such as negative or positive affect, risky sexual behavior, or substance use. Further research on optimal designs of EMA interventions for MSM is warranted.

## Introduction

### Ecological Momentary Assessment 

Developed originally from the field of personality and social psychology, ecological momentary assessment (EMA) is a research design with methodological components that allow researchers to measure experiences as close to that moment as possible and within the participant’s natural environment [1]. Methodological strategies in EMAs have included prompting participants at various time intervals using self-report surveys or triggers specific to locations, events, or both [2]. Researchers have indicated that recall-based self-reports can be inaccurate or unreliable measurements of participants’ actual lived experiences [2]. The goal of EMA is to minimize retrospective recall issues and enhance researchers’ ability to measure lived experiences of people in the moment [1].

The ability to measure and potentially intervene in lived experiences in the moment is especially important to impact dysfunctional thoughts, capture psychological distress, or even intervene in harmful behavior [2]. In addition, one of the key benefits and goals of EMA is to provide high levels of ecological validity, or validity that comes from collecting data and implementing an experiment in a participant’s natural setting in real-world contexts. High ecological validity can enhance the ability for research findings to be applied to real-world scenarios, increasing the likelihood of generalizability [3]. Research has also found EMA methods to outperform traditional paper-pencil measurements in the ability to determine needs of clinical interventions more precisely. A primary reason that EMA measurements outperform traditional paper-pencil measurements is that repeated measurements minimize the effect of participants’ current state on results [4]. Finally, technology-based interventions incorporating EMA methods have shown promise in terms of feasibility and acceptability of enhancing intervention outcomes [5].

### Men Who Have Sex With Men

Men who have sex with men (MSM) have been found to show high rates of both psychological distress and engagement in various risky behaviors [6]. Specifically, studies have found MSM to endure higher levels of depression, anxiety, substance use, and risk of contracting HIV [7,8]. EMAs have been used among MSM in daily diaries since 2007 [7] and have evolved tremendously into the realm of internet use [9], smartphone technology [10], and interventions [11]. Use of EMAs among MSM is a growing area of research. EMAs have been shown to be highly effective in reaching people who have a history of substance use or other risky behavior, due to the minimization of stigma and enhancement of self-control over privacy, confidentiality, and anonymity [10,12,13].

### Scoping Review

The primary purpose of a scoping review is to synthesize current literature surrounding a topic area. Thus, the synthesis produced from a scoping review acts as a summary of available literature, a means to identify key concepts, and a precursor to a systematic review [14]. To the best of the authors’ knowledge, neither a scoping review nor a systematic review has yet been produced on the topic of EMA use among MSM, due to the limited literature surrounding the topic. As a result, the authors intended to conduct a scoping review by applying the PRISMA-ScR (Preferred Reporting Items for Systematic Reviews and Meta-Analyses-Extension for Scoping Reviews) [15], as a contribution toward a future systematic review based on an increase in literature. Authors also acknowledge the limitations of a scoping review, mainly, an inability to make quality assurances, a lack of strong validity, and an inability to hypothesize based on the review.

### Scoping Review for EMAs Among MSM 

Despite the benefits and clinical implications of using EMA methods for at-risk populations, there have been no reviews compiling the literature of how EMAs have been used among MSM. One growing method for synthesizing theoretical and empirical evidence in the literature is the scoping review [16]. Scoping reviews are considered as means to describe key findings across literature, identify gaps in research, and inform the design of future research studies [16]. Two major benefits of conducting a scoping review are the ability to examine the breadth of the topic of EMA methodologies that are applied to MSM, specifically, within interventions, and the ability to identify knowledge gaps and future directions for the expansion of this area of research. The aims of this study were to: (1) synthesize the literature across fields on how EMA methods have been used among MSM, (2) better understand the feasibility and acceptability of EMA interventions among MSM, and (3) to inform designs for future research studies on best evidence-based practices for EMA interventions.

## Methods

### Eligibility Criteria

We identified several eligibility criteria that needed to be met for a publication to be included in this scoping review. The eligibility criteria included MSM samples; EMA interventions or assessments or determinations of EMA’s efficacy, acceptability, and feasibility; a publication date within the past 5 years; peer-reviewed studies conducted in the United States; and quantitative data analyses.

### Information Sources

**Figure 1:**
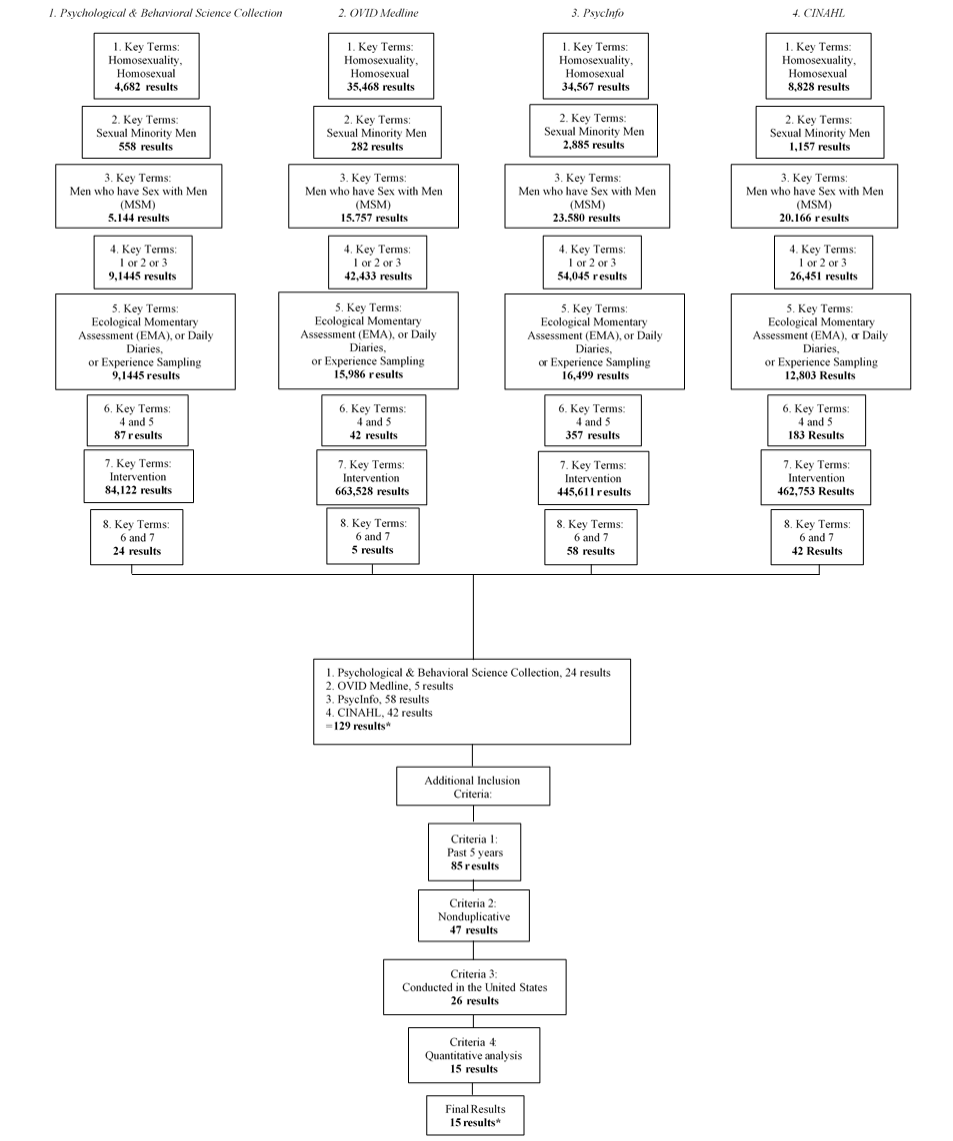
Flow diagram of study selection process. CINAHL: Cumulative Index to Nursing and Allied Health Literature; *Peer-reviewed articles.

We chose 4 prominent databases to retain studies from: Ovid Medline, which focuses on biomedical scholarly literature; Psychological and Behavioral Science Collection, which focuses on mental processes and emotional and behavioral experiences; PsycInfo, which focuses on behavioral and social science research; and Cumulative Index to Nursing and Allied Health Literature, which provides access to health research, specifically, in nursing and other allied health. EMAs have predominately been applied to behavioral or psychological health [17,18] and physical health [19,20]. Therefore, these databases were determined as most relevant and applicable to the specific topic of this scoping review (Figure 1).

### Keywords and Search Process

To ensure that we conducted an inclusive review of the literature across databases, we used multiple search terms for our target populations. Step 1 involved searching the terms “homosexuality” and *“*homosexual.*”* In step 2, we used the search term *“*sexual minority men.” Finally, in step 3, we used the search terms “men who have sex with men” and “MSM.” In step 4, we combined all these terms to achieve the most comprehensive review of literature pertaining to our target population of MSM. In step 5, we added the keywords “ecological momentary assessment” or “EMA,” “daily diaries,” and “experience sampling” to identify all EMA-related literature from these databases. In step 6, we combined our MSM search terms with the EMA terms to narrow down to only the most directly relevant articles. Upon reaching step 7, we applied the term “intervention” to further narrow the articles included in this review. Step 8 was comprised of combining all search terms from our MSM terms, EMA terms, and intervention terms, which resulted in a total of 129 articles.

### Inclusion Criteria, Exclusion Criteria, and the Iterative Process

Once we had the initial studies from our database searches, we combined all the study titles and previewed the articles. Articles were further narrowed based on 2 additional inclusion criteria and 2 additional exclusion criteria; studies were excluded if they were not published in the past 5 years and if they were duplicative across databases. Studies were included if they were conducted in the United States and included quantitative data and analyses (Figure 1).

As suggested by the clarity of guidelines in scoping reviews [21], our search and review were conducted in an iterative manner over time. We conducted our first search in January 2020, our second search in May 2020, our third search in September 2020, and the last search in March 2021, as presented, to examine quarterly changes in the literature.

### Analysis

Modeling after the Centers for Disease Control and Prevention (CDC) Compendium of Evidence-Based Interventions (EBIs) [22], we primarily used 8 distinct criteria to review the final set of eligible publications [9,11,13,23-34]. The EBI criteria and best practices in the compendium, developed and defined by the CDC’s Prevention Research Synthesis project, posit a series of systematic review components for interventions. EBI criteria have been shown to generate significant effects and strong evidence of efficacy in HIV-related outcomes [22]. Based on the compendium of EBIs, our review criteria included (1) citation, sample size, and duration of study; (2) location; (3) random assignment (yes or no); (4) key aspects of the intervention; (5) follow-up (yes or no); (6) occurrence of follow-up after intervention; (7) rate of retention; and (8) rate of engagement (Multimedia Appendix 1). We also conducted a secondary analysis comprised of additional review criteria: recruitment strategy, description of intervention and comparison of arms, specific measurement tools, outcomes of interest, and outcome results (Multimedia Appendix 2).

## Results

### Risk of Quality in Individual Studies

Using the CDC compendium of best evidence-based risk reduction for individual-level interventions, we evaluated the strength of each study. None of the studies reviewed met full criteria for best standards of risk reduction in individual-level interventions. However, we continued evaluating based off CDC compendium criteria to determine study designs, intervention elements, and highest standards currently achieved.

### Study Selection

A total of 129 articles were identified in the preliminary search: 24 from the Psychology and Behavioral Science Collection, 5 from OVID Medline, 58 from PsycInfo, and 42 from Cumulative Index to Nursing and Allied Health Literature. Among these 129 articles, we excluded 114 studies for not meeting additional screening criteria. These 4 criteria that the studies had to meet were: (1) the study was published in the past 5 years, eliminating 44 studies; (2) not a duplicative study, eliminating 38 studies; (3) a US-based study, eliminating 12 studies; and (4) the study used a quantitative analysis, eliminating 9 studies. The qualifying criteria led to a final set of 15 studies in this review [9,11,13,23-34].

### Sample Sizes

The average sample size across the 15 studies reviewed was 63.5 (SD 31.85). The CDC recommends that each sample is greater than 50 participants per study arm. Among the 15 studies reviewed, 10 (67%) had sample sizes >50, and 4 (27%) had sample sizes ≥100 [9,31,33,34]. The largest sample size was 120, in a study that had a single intervention arm [31]. Of 15 studies, 4 (27%) had multiple intervention arms [11,26,27,30].

### Demographics

The only 2 demographic measurements reported by all 15 articles were race/ethnicity and age [9,11,13,23-34]. Within the total sample of 952 participants that was developed from a composite of all article samples, the majority of participants (476/952, 50%) were white, and this sample had a mean age of 38.75 years (SD 8.5). Of 15 studies, across 9 studies (60%), income was also reported, with the majority of the composite sample making <$40,000 annually [9,11,13,25,26,31-34]. The final demographic measurement that was majorly reported, across 12 of the 15 studies (80%), was education; the majority (420/952, 44.1%) of this composite sample had at least some college education [9,11,13,24-26,28,29,31-34]. A detailed synopsis of demographics can be found in Multimedia Appendix 3.

### Key Aspects of the Interventions

From this review, we determined that multiple studies had similar key aspects across the interventions implemented. First, 7 of the 15 studies (47%) focused on multiple types of substance use, including nicotine use, alcohol use, or other substance use (eg, cocaine, methamphetamine, and cannabis). Second, affect and stigma were discussed as primary outcomes for 5 of 15 studies (33%) [11,28-30,34] and secondary outcomes for 3 additional studies of the 15 studies (20%) [27,32,33]. Among 15 studies reviewed, 6 (40%) of them focused on examining feasibility or acceptability of EMA methodologies within the intervention [11,13,23,25,31]. Finally, 60% (9/15) of the studies focused on sexual behavior among men who have sex with men [9,11,23,25,28,30,32-34].

### Random Assignment

Random assignment occurred in 2 of the 15 studies (13%) included in our review [11,26]. The CDC recommends random assignment as a gold standard, to rule out biases in a systematic way across multiple intervention arms. Since the majority of the studies (11/15, 73%) included in this review had only 1 arm, random assignment was not implemented [9,13,23-25,28,29, 32-34]. In the remaining 2 studies that had nonrandomized designs with multiple arms, one study used a quasi-experimental design implementing clinical cutoffs for hypersexuality to determine group membership [30], while the other study assigned groups based on whether participants were recruited in-person or online [9]. Random assignment was considered as potentially unethical in many of these studies due to their focus on substance use and sexual behavior; thus, a quasi-experimental design was better suited.

### Description of Intervention and Comparison Arms

Across the articles, there were a wide variety of intervention strategies used for implementation, for example, impacting the duration, mechanism for the intervention, and tasks for intervention participants. The average duration of the intervention was 31.5 days; the shortest intervention was a single-day cross-sectional survey [13], and the longest was a 90-day intervention [31]. Interventions were conducted primarily via mobile devices (14/15, 94%) [9,11,23-34]. Among the interventions conducted through mobile devices, 11 of 14 studies (79%) used texting prompts [13,28-30]. Tasks to be completed were predominately surveys (10/15, 67%) [11,13,24-31] administered through text message (6/15, 40%) [11,24-27,31]. The second-most used modality for data collection was daily diaries (5/15, 33%) [9,23,32-34]. The CDC recommends a clear intervention description, which we found across all the included studies [22].

### Follow-up and Occurrence of Follow-up

Follow-up assessments were administered in 2 studies of the 15 (13%) [11,31], one of which included follow-ups at 3 different time points: 60 days, 90 days, and 120 days [31], while the other followed up with participants after 4 weeks [11]. According to the CDC compendium, there should be a follow-up, and it should, specifically, occur more than 30 days after completion of the intervention [22].

### Rate of Retention

Rate of retention was measured in 33% (5/15) of the included studies [11,25-27,31]. Among those that measured retention, the average retention rate was 77.58%, the lowest retention rate was 29.2% [31], and the highest retention rate was 93% [26]. For a high-quality intervention, the CDC recommends a 70% study retention rate [22].

### Rate of Engagement

Of the 15 studies, 13 (87%) reported rate of engagement [9,13,23-29,31-34]. Engagement rate was defined as an overall rate of completion for text or online surveys, text prompts, or daily diaries, depending on the study modality and was reported consistently across all studies. The average overall engagement rate was 76.93%, the lowest overall engagement rate was 37.3% [9], and the highest was 98.7% [13]. Engagement is a key component of retention, and the 70% retention rate is recommended as the benchmark for an acceptable engagement rate [22].

### Location

The location of the catchment areas and study sites varied. The majority of studies (9/15, 60%) were conducted on the East Coast [9,13,23,25,28,29,32-34]. Within those conducted on the East Coast, the majority of these studies (5/9, 56%) were concentrated in the Northeast [9,23,32-34]. The second-most researched area was the West Coast (4/15, 27%) [11,26,27,31], with a focus on San Francisco (3/15, 20%) [26,27,31]. Of the 15 studies, 1 (7%) was conducted in the Northwest [24] and 1 (7%), in the upper Midwest [29].

### Recruitment Strategies

Of the 15 studies, 12 (80%) used more than 2 recruitment strategies. Of those that used at least 2 recruitment strategies, 14/15 (93%) studies paired their strategies with social media (e.g., Instagram or Facebook) [9,11,23-34]. Of the 15 studies, 3 (20%) used cohorts from larger or alternative study sites [11,13,30], one of which used multiple recruitment strategies, including social media [30]. The most popular recruitment strategy that was paired with social media was the use of community-based organizations with in-person recruitment (6/15, 40%) [11,25-27,30,31].

### Specific Measurement Tools

The majority of the studies (10/15, 67%) were conducted with EMA surveys [11,13,24-31]. Of these 10 studies, 2 (20%) used the same scale to measure affect, the Positive and Negative Affect Scale [9,30]. Of these 10 studies, 2 others (20%) administered the Difficulties with Emotion Regulation Scale to measure emotion dysregulation [28,29]. Many studies used questions such as how many partners a participant engaged in sex with over the past 24 hours (9/15, 60%) [9,11,23,25,30-34], how many standard alcoholic drinks a participant consumed in the past 24 hours (6/15, 40%) [9,24,26,32-34], and what types of drugs were used over the past 24 hours (11/15, 73%) [9,23,24,26,27,29-34].

### Outcomes of Interest

There were 3 prominent outcomes of interest across the included studies: risky sexual behavior, substance use, and acceptability. Of the 15 studies, 7 (47%) measured substance use status, including use of nicotine, alcohol, and other nonprescription drugs [9,11,24,27,29,33,34]. Of the 15 studies, 6 (40%) measured sexual behavior, especially, risky, unprotected sexual behavior defined by condomless sex or sex with partners who were of unknown HIV status [11,23,30,32-34]. Of the 15 studies, 4 (27%) assessed the acceptability of EMAs implemented in an intervention by measuring response rates, completion rates, and study retention rates [9,25,26,31].

### Outcome Results

Intervention studies using EMA methods have demonstrated success in longitudinally measuring substance use, compared with studies that relied on non-EMA measurements such as timeline follow-back surveys [9,23,32]. Additionally, EMA methods generated greater acceptability than other methods: daily diaries had high rates of response completion. The highest response rate was 97.3% [9,34], and the lowest was only 84% [23].

## Discussion

### Principal Findings

In our scoping review, we aimed to provide an overview of the growing literature on a relatively novel measurement: ecological momentary assessment (EMA). We found that among men who have sex with men (MSM), the majority of EMAs have been used to intervene on risk-taking behaviors such as alcohol and drug use or unprotected sex with multiple partners. Although risk-taking behaviors have often been stigmatized, the use of EMA through smartphone technology has been seen as a highly effective way to safely assess risk-taking behaviors [10,12,13]. Overall, EMA was seen as an acceptable and feasible method, with daily diaries as the most acceptable tool [9,23,32-34] to collect the experiences of MSM. A unique facet of MSM research was the successful use of recruitment strategies beyond technology-based recruitment, which included assistance in initiation and engagement from community-based organizations [11,25-27,30,31]. This scoping review was used as a synthesizing method with a wide array of review dimensions and criteria such as quantitative interventions among MSM. This allowed our results to provide a comprehensive set of typological frameworks that may be useful in designing and implementing an EMA-integrated intervention for behavioral change. Basing typographic dimensions off preexisting frameworks offered by the CDC [22], we incorporated the most salient components for intervention research. This allowed for a better assessment of the strength of existing EMA interventions among MSM. Also, we conducted 4 time points of literature searches (ie, January 2020, June 2020, September 2020, and March 2021), for inclusion of more studies, which increased the comprehensiveness of this scoping review.

Our study determined that there were limited psychometrically sound EMA measurements that were fully validated. Given the growing research attention on EMAs within the context of behavioral intervention, future studies may aim to develop and validate EMA measurements. EMA has been widely used as a just-in-time assessment and monitoring tool, but it also can be a great measurement resource to predict behavioral outcomes. We suggest future research should focus on developing predictive models and analytic methods, using intensive longitudinal data from EMAs to understand behavioral changes or outcomes over time. In terms of an analytic perspective, since EMAs lead to extensive longitudinal data, the risk of missingness and the handling of missing data will become more prevalent. Therefore, studies on appropriate analytic approaches to manage missing data from EMAs will be essential.

Although EMAs may reduce recall bias, due to the repetitiveness of measurement, they can also increase participant bias and burden [35,36]. Future research should take into consideration EMA designs that are engaging but protective of data anonymity and confidentiality, to prevent participant biases such as the social desirability effect and the halo or devil effect. To avoid priming of such participant biases, details and information in EMA-based interventions should be presented in a judgment-free manner. In order to reduce psychological reactions, future research should consider developing EMAs as self-motivated mechanisms, with use options such as event-contingent prompts, daily diaries, text prompts, or other mechanisms. Additionally, future studies should examine putative mechanism factors such as resilience and social support, to develop a comprehensive, integrative intervention program for MSM [37].

### Limitations

There were limitations imposed by the scope and design of the study. First, the inclusion of the major library databases focused on studies most relevant to the population and methodological strategy of interest, but we excluded other minor library databases. Therefore, a future direction may include an inclusion of minor databases in the review for EMA interventions among MSM. Second, many of the studies included in this review were feasibility and acceptability tests as well as pilot studies with inconsistent assessments of outcomes, thus minimizing the effectiveness of a meta-analysis or systematic review. Although we presented results from a scoping review to provide an overview and the state of EMA in behavioral medicine, future research may conduct a systematic review or meta-analysis, as the prevalence of empirical evidence from randomized controlled trials using EMAs in this area is likely to increase [38].

### Conclusions

Leveraging evidence-based intervention designs with validated ecological momentary assessments can advance our understanding of factors and processes in behavioral changes and health outcomes. These approaches can be further empowered through technology-based behavioral medicine and social medicine. In this scoping review paper, we provided a typology of EMA-based intervention research that was designed to promote health behavior and psychological well-being. Advancements in psychometric tests to validate EMAs will be critical. As the empirical evidence and theories in this field are emerging, we hope our review offers some guidance and synthesis of the literature to develop and evaluate technology-based EMA health interventions.

## Appendix

Multimedia Appendix 1 Primary scoping review analysis.

Multimedia Appendix 2 Secondary scoping review analysis.

Multimedia Appendix 3 Demographics.

## Abbreviations

CDC: Centers for Disease Control and Prevention
EBI: evidence-based intervention
EMA: ecological momentary assessment
MSM: men who have sex with men
PRISMA-ScR: Preferred Reporting Items for Systematic Reviews and Meta-Analyses - Extension for Scoping Reviews
