# Supplementary figures and images for: Ecological Momentary Assessment and mHealth Interventions Among Men Who Have Sex With Men: Scoping Review
Source: J Med Internet Res. 2021 Aug 3;23(8):e27751. doi: 10.2196/27751 (PMC8371491; doi:10.2196/27751)

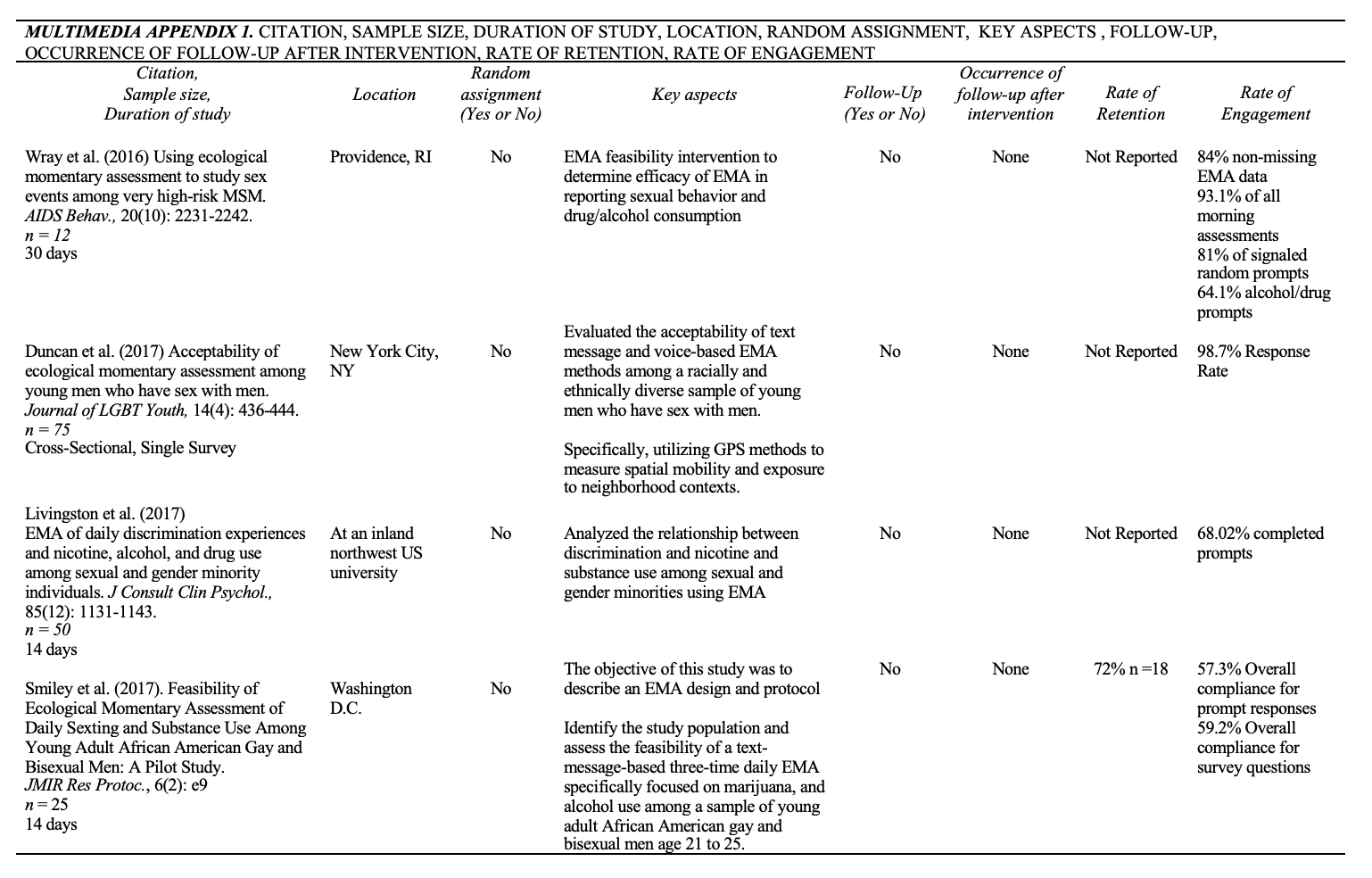


**
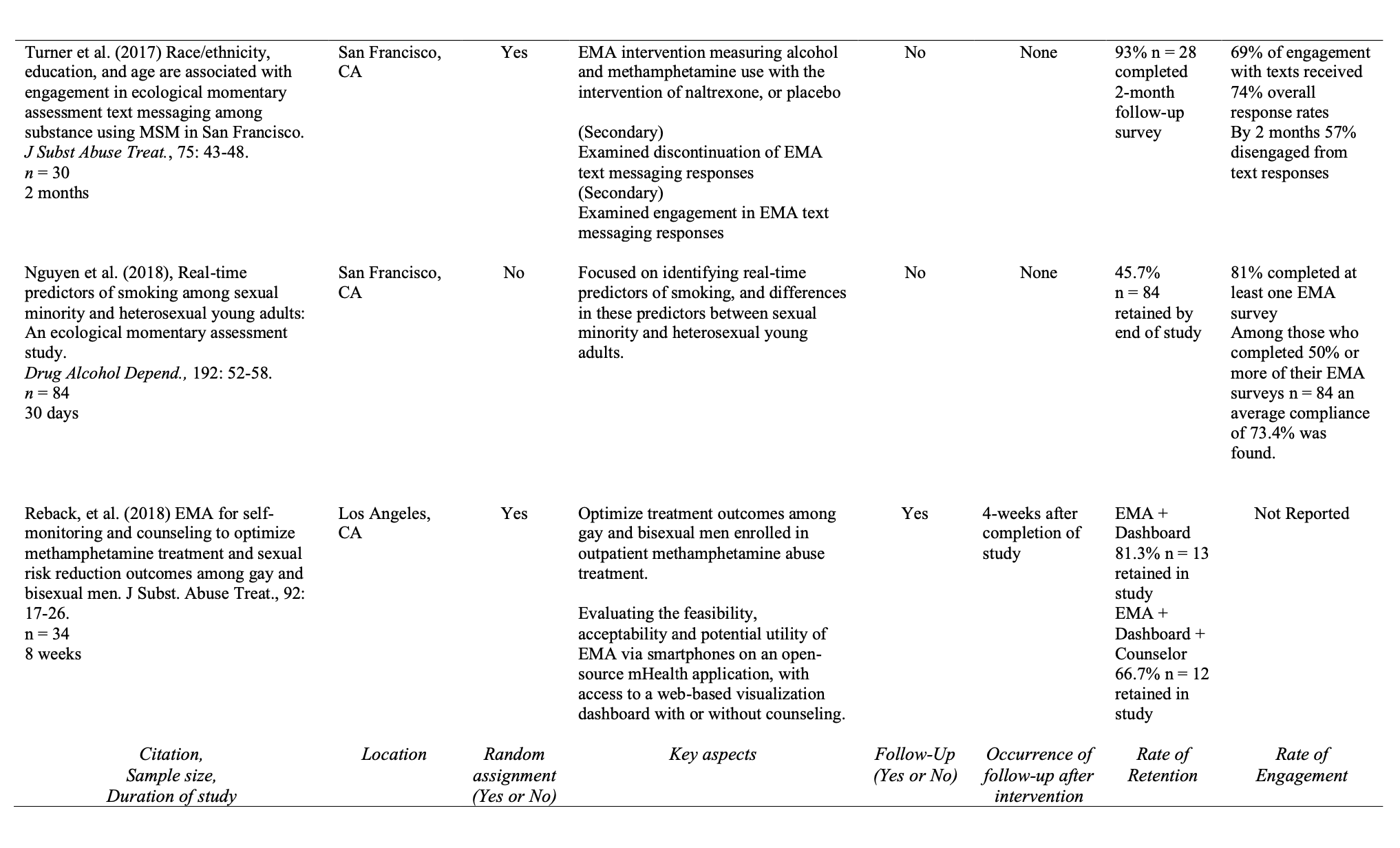
**

**
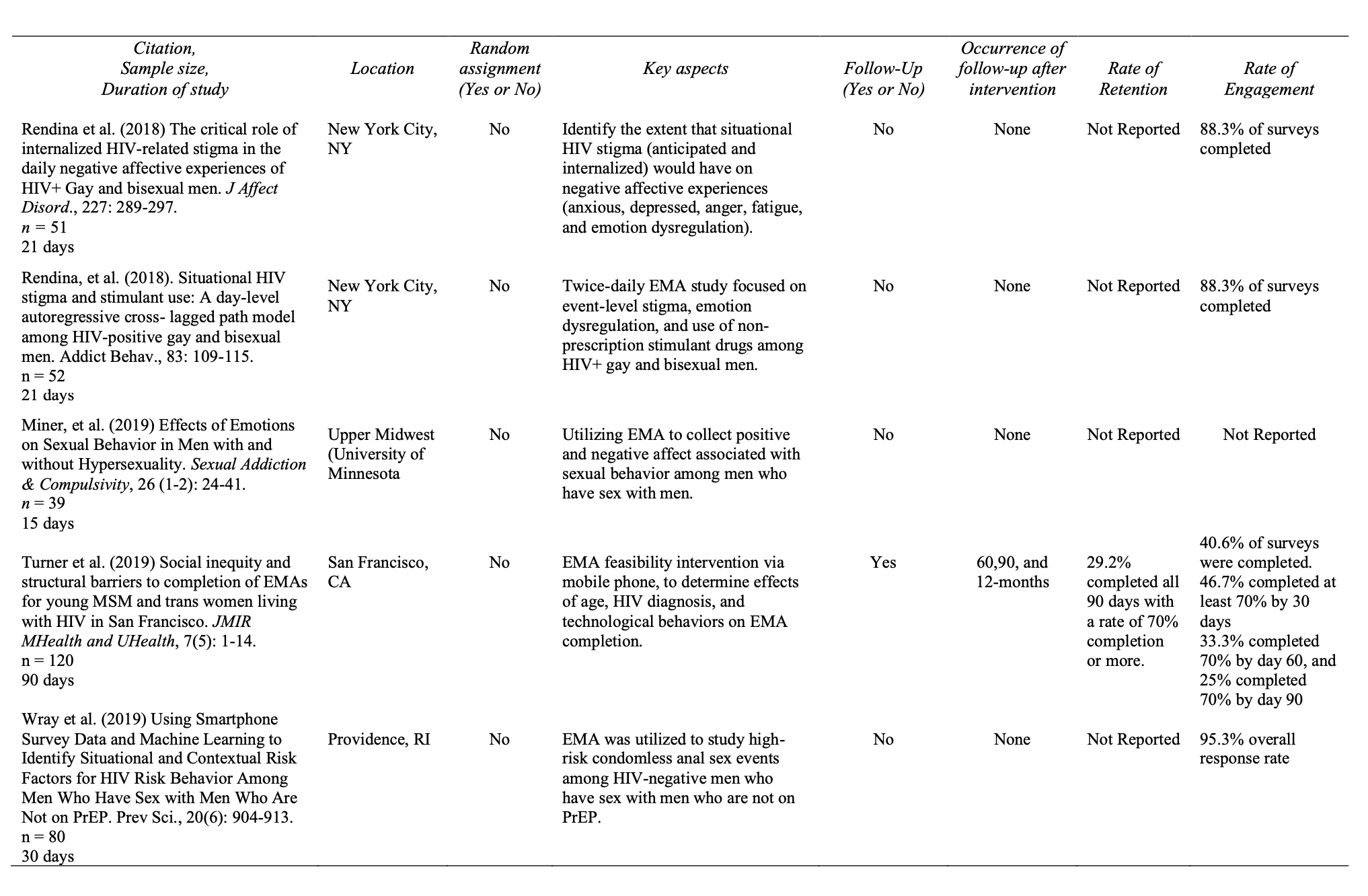
**

**
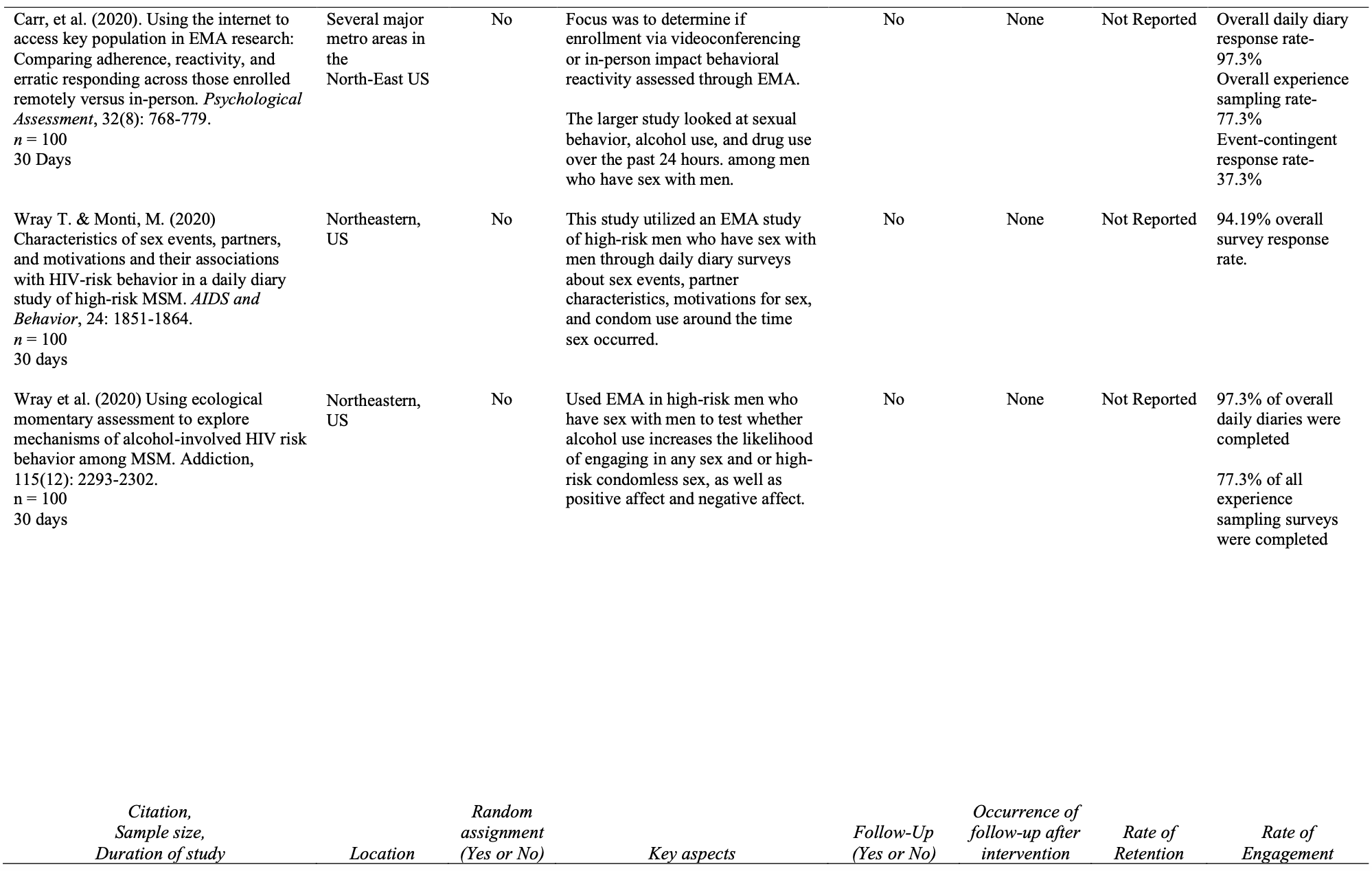
**

Supplement: Multimedia Appendix 1 [file jmir_v23i8e27751_app1.docx]

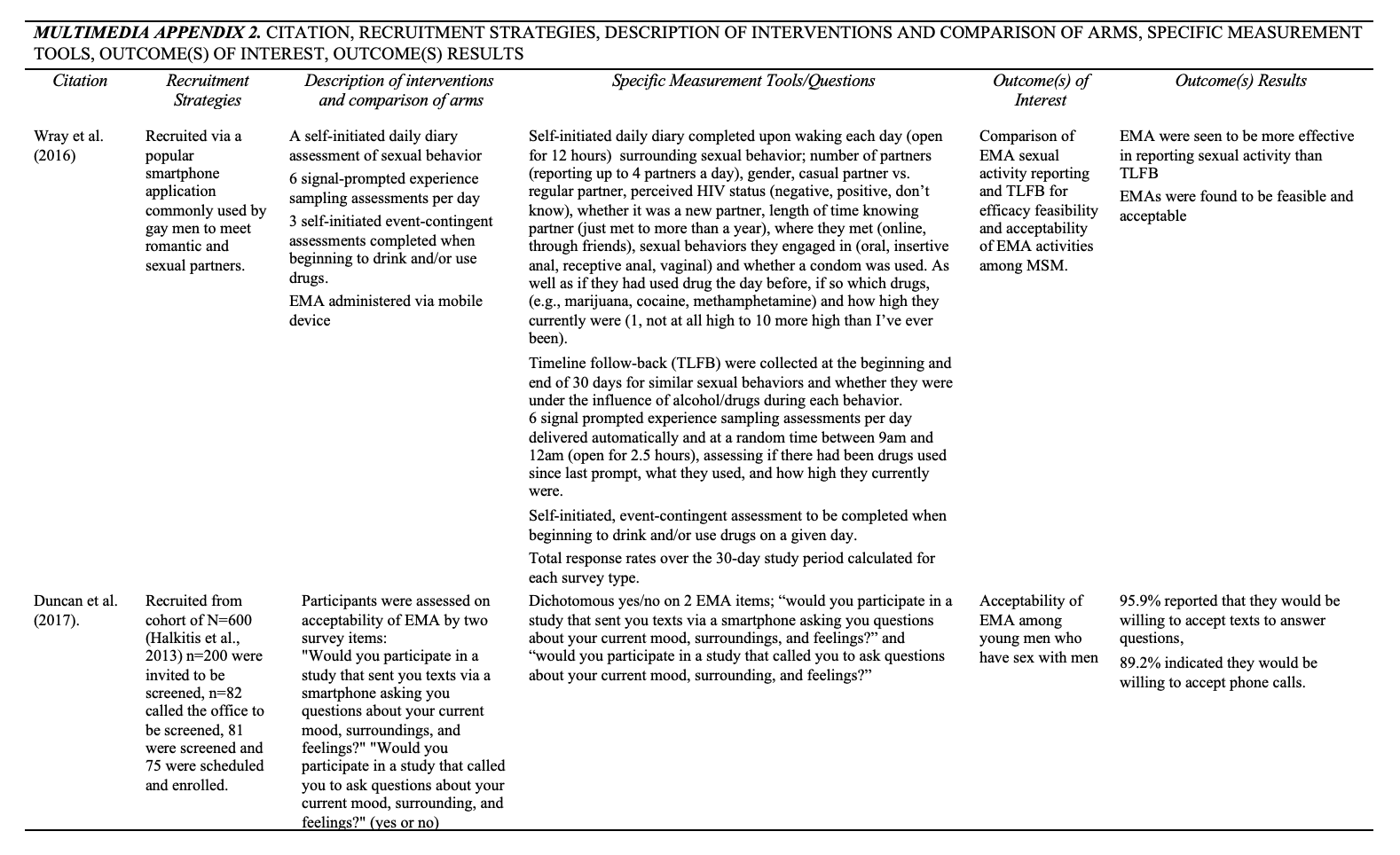


**
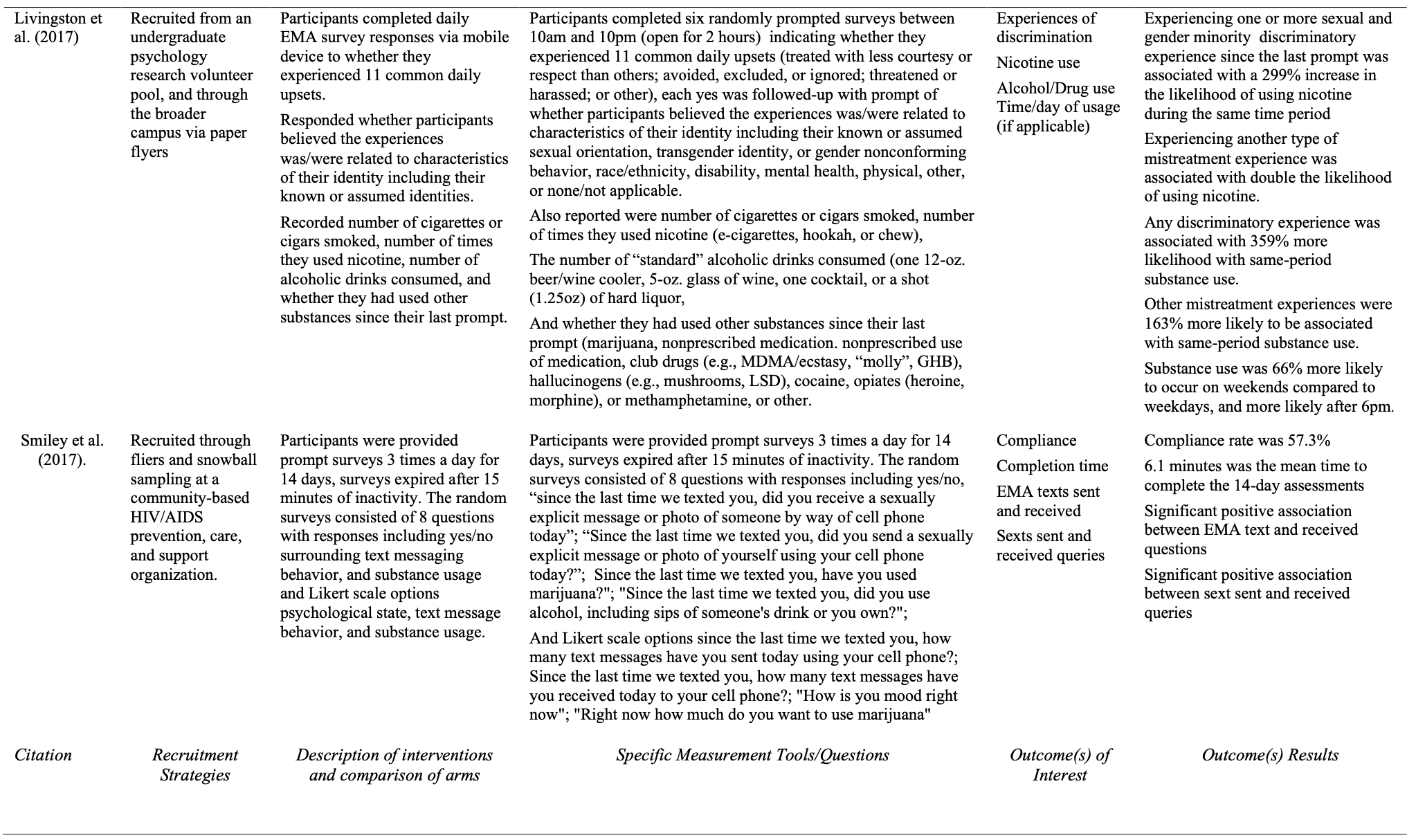
**

**
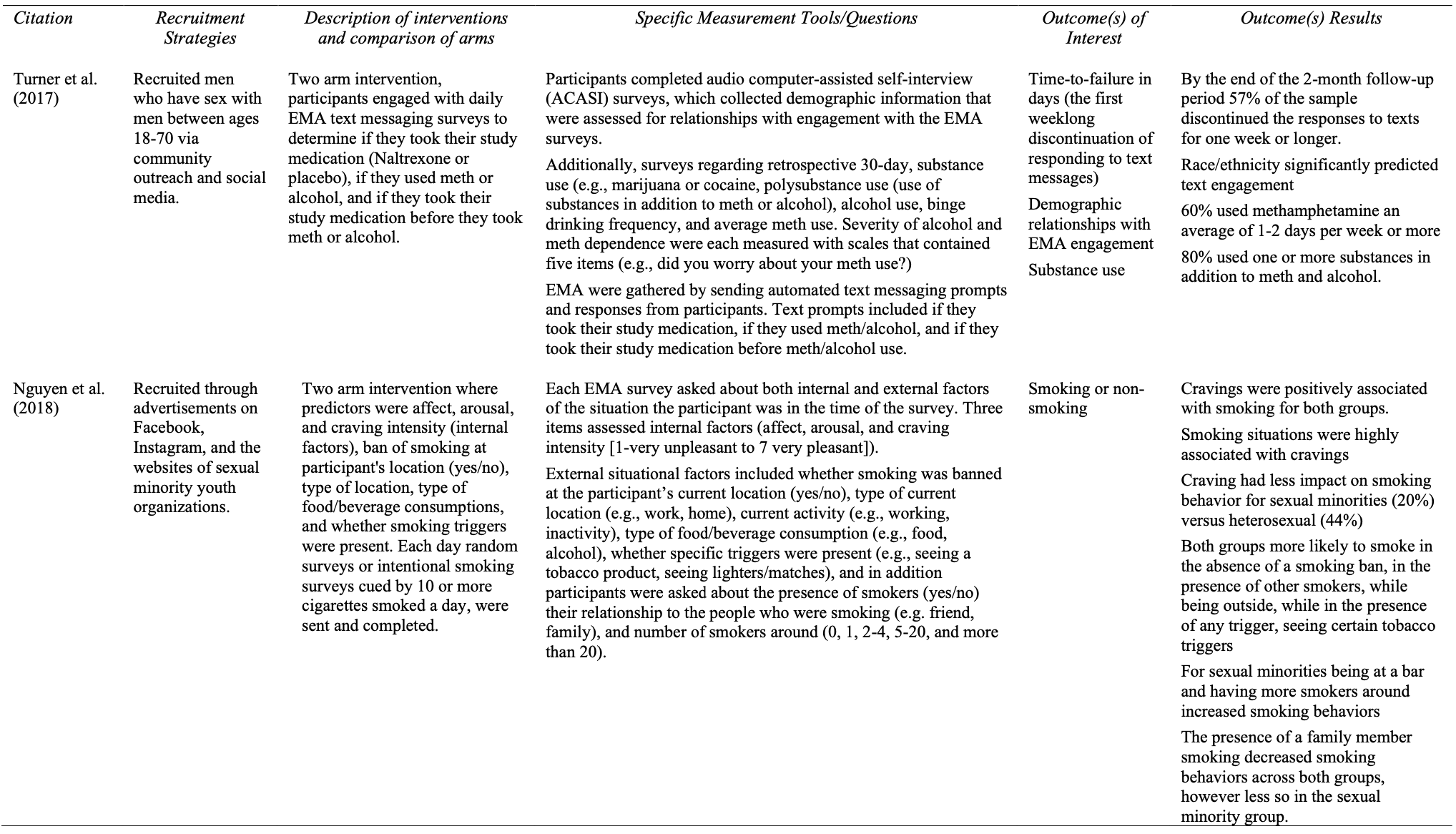
**

**
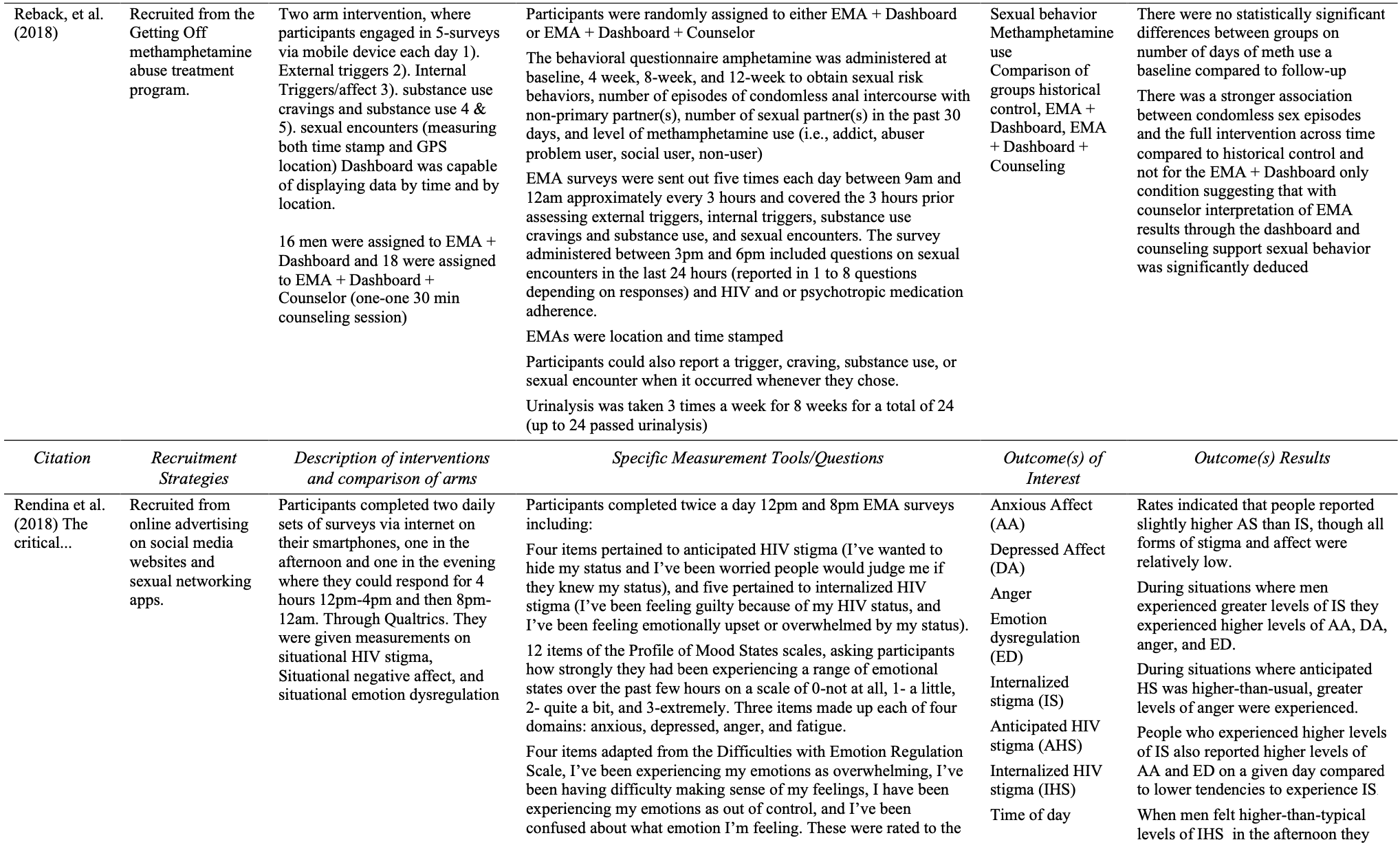
**

**
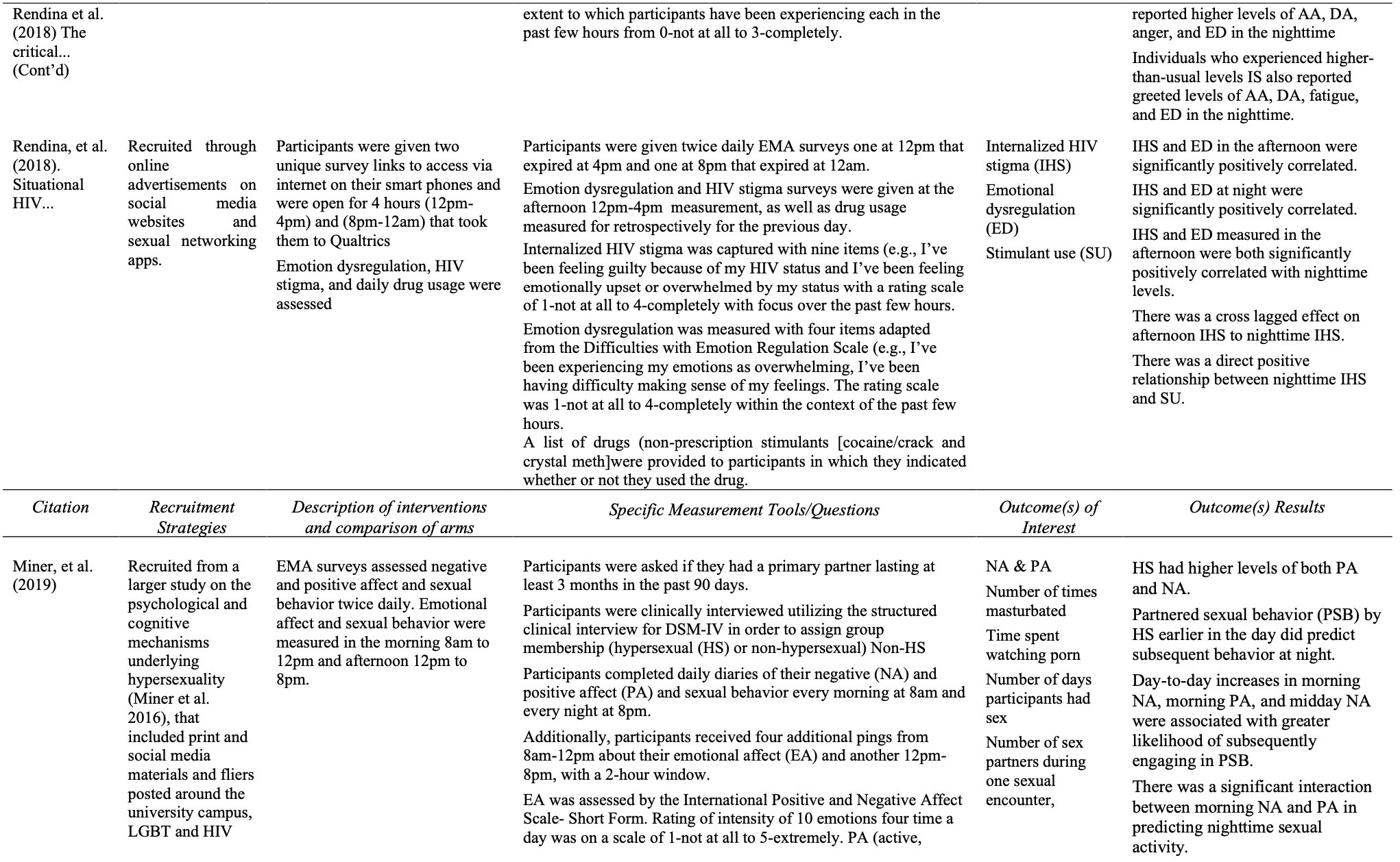
**

**
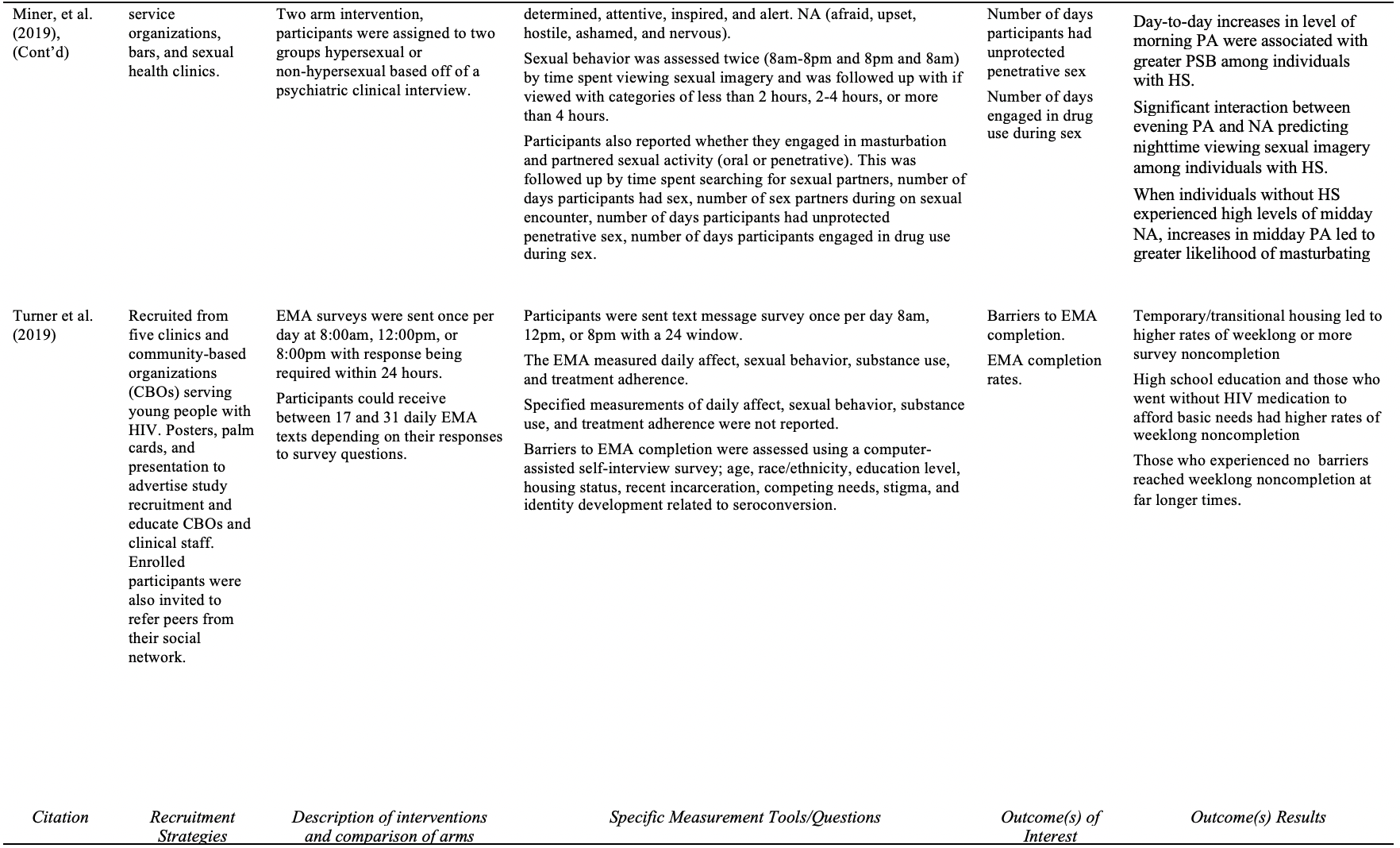
**

**
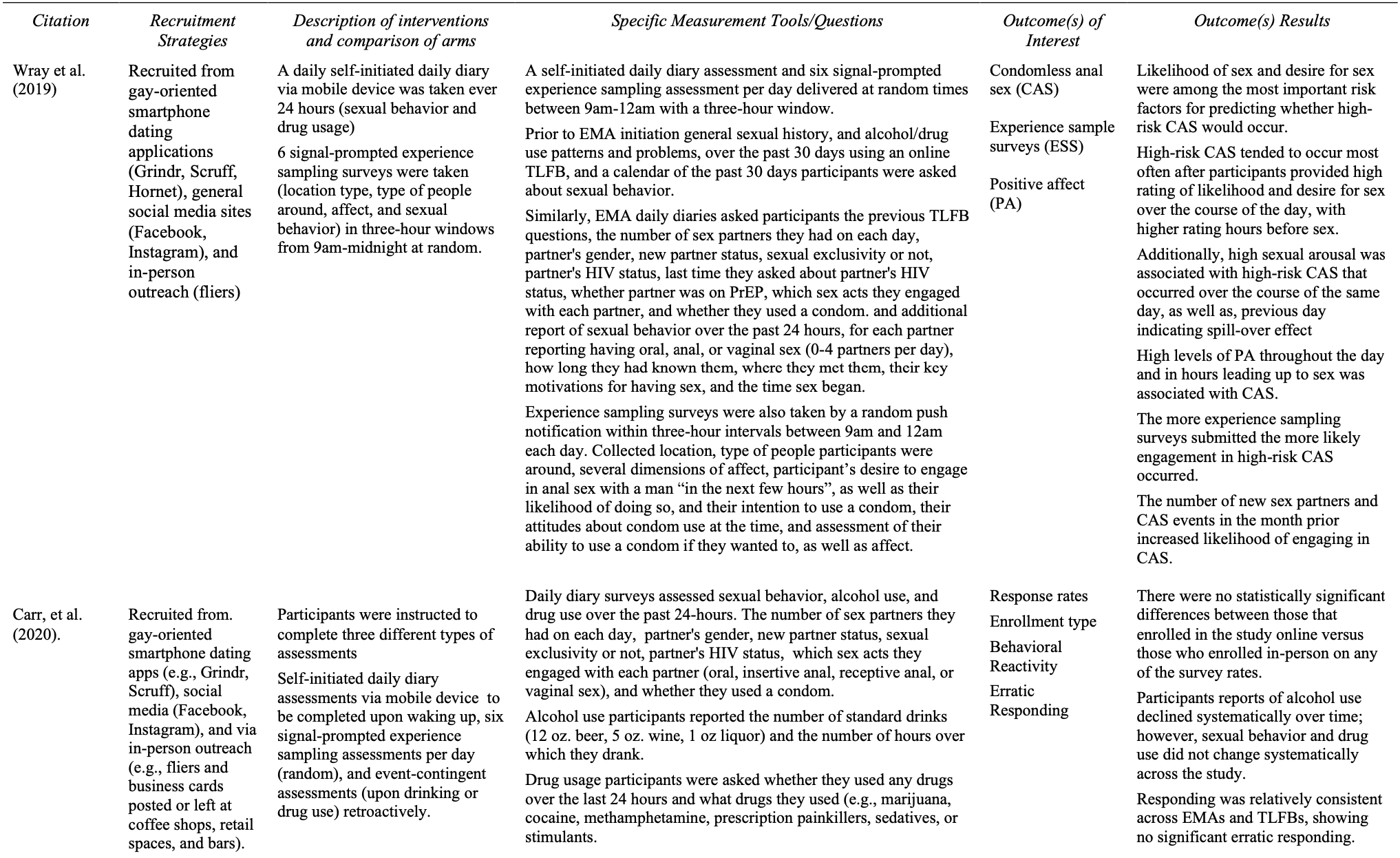
**

**
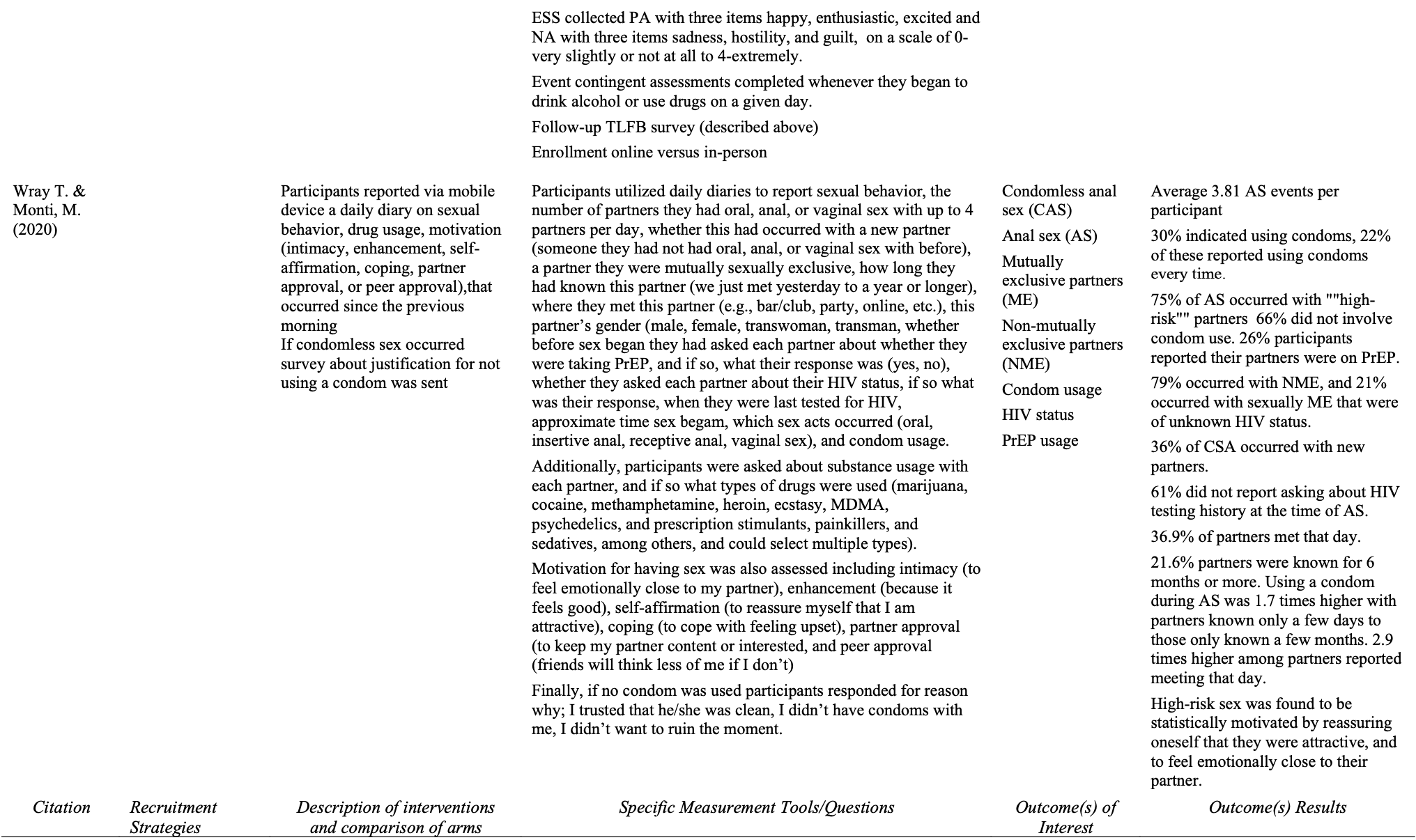
**

**
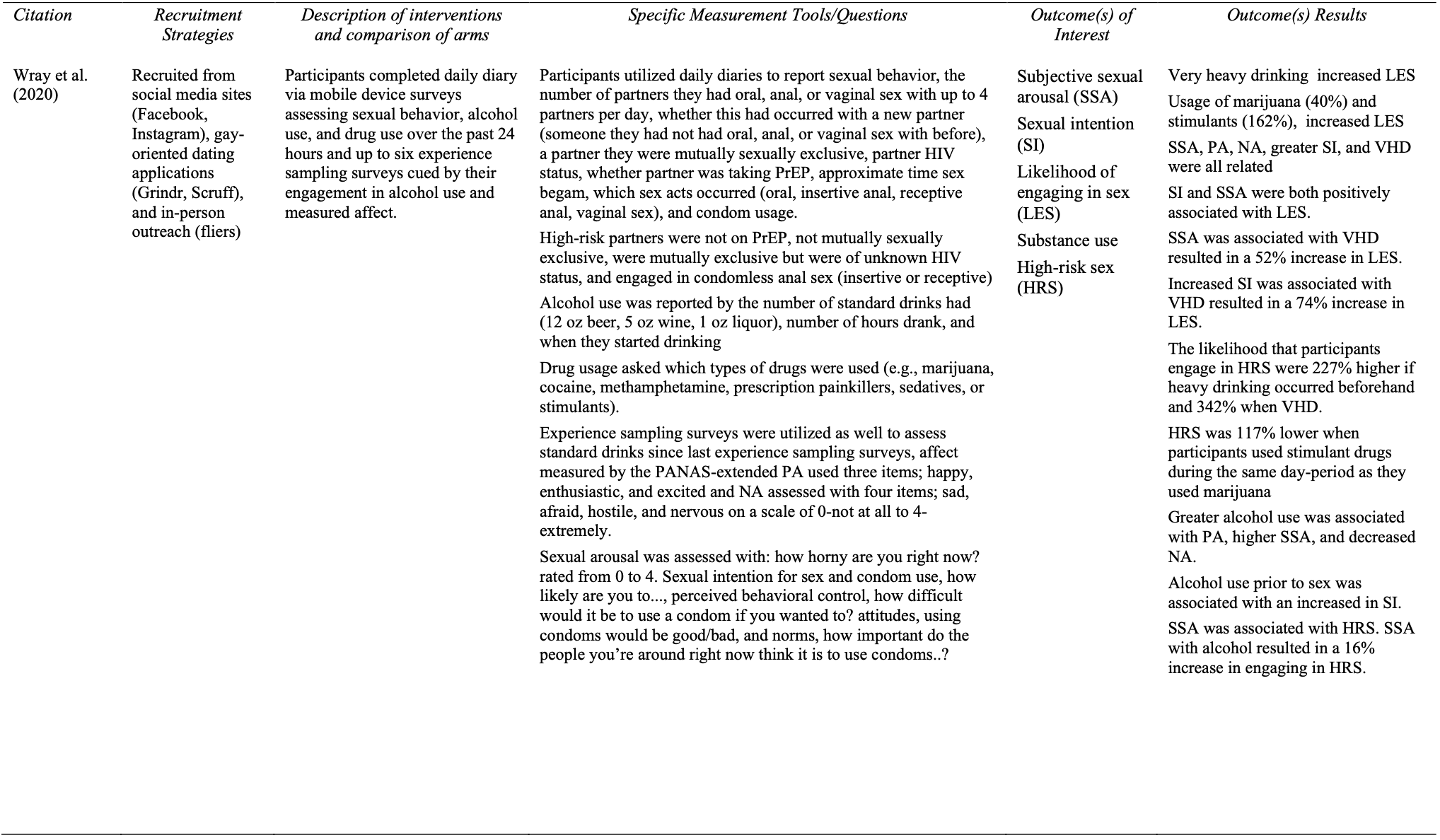
**

Supplement: Multimedia Appendix 2 [file jmir_v23i8e27751_app2.docx]
