# Supplementary material for: Ecological Momentary Assessment and mHealth Interventions Among Men Who Have Sex With Men: Scoping Review
Source: J Med Internet Res. 2021 Aug 3;23(8):e27751. doi: 10.2196/27751 (PMC8371491; doi:10.2196/27751)
Supplement: Multimedia Appendix 3 [file jmir_v23i8e27751_app3.docx]

| ***Multimedia Appendix 3: Demographics*** | | | | | | | | | | | |
| --- | --- | --- | --- | --- | --- | --- | --- | --- | --- | --- | --- |
|  | | | **Race/Ethnicity *n* (%)** | **Age µ (SD**) | | | | **Income *n* (%)** | **Education *n* (%)** | | |
| Wray, et al., 2016 | | | Hispanic 2(16.7)  White 10(83.3)  Total 12(100) | 30 (8.8) | | | | Not Reported | Not Reported | | |
| Duncan, et al., 2017 | | | White 17(22.9)  Black/African American 25(33.8)  Hispanic/Latino 21(28.4)  Asian/Pacific Islander 4(5.4)  Multiracial/Other 6(8.1)  Not Report 1(1.4)  Total 74(100) | 22 (0.6) | | | | < $15,000 37(50)  $15,000-  $35,000 27(36.5)  > $35,000 8(10.8)  Not Reported 2(2.7) | High School or less 45(60.8)  Some College/Tech School 8(10.8)  College Degree 21(28.4) | | |
| Livingston, et al., 2017 | | | White 42(84)  Native American/ 1(2)  Alaskan Native  Multiracial 7(14)  Total 50(100) | 21.82 (4.7) | | | | Not Reported | High School 5(10)  Some College 37(74)  Associate’s Degree 1(2)  Bachelor’s Degree 6(12)  Graduate or Professional Degree 1(2) | | |
| Smiley, et al., 2017 | | | Black/African American 21(84)  Multiracial 4(16)  Total 25(100) | 23.48 (1.5) | | | | < $35,000 15(60)  > $35,000 8(32)  Unsure 2(8) | High School of less 4(16)  Some College 21(84) | | |
| Turner, et al., 2017 | | | High Engagement  MSM of Color 10(77)  White MSM 3(23)  Total Subsample 13(100)  Low Engagement  MSM of Color 2(11.8)  White MSM 15(88.2)  Total Subsample 17(100)  Total 30(100) | 39.38 (9.9)  45.76 (8.0) | | | | < $40,000 10(77)  > $40,000 3(23)  < $40,000 14(82.4)  > $40,000 1(5.9)  Not Reported 2(11.7) | College or more 13(100)  Less than college 4(23.5)  College or more 13(76.5) | | |
| Nguyen, et al., 2018 | | | Non-Hispanic White 33(39.3)  Non-Hispanic Black 3(3.6)  Asian 20(23.8)  American Indian/ 1(1.2)  Alaskan Native  Hawaiian Pacific Islander 1(1.2)  Hispanic 15(17.8)  Other/Multi-Race 11(13.1)  Total 84(100) | 23.08 (2.3) | | | | Not Reported | Not Reported | | |
| Reback, et al., 2018 | | | White 12(35.3)  Hispanic/Latino 11(32.3)  Black/African American 7(20.6)  Multi/Other 4(11.8)  Total 34(100) | 40.6 (9.3) | | | | <$15,001 28(82.4)  $15,001- 3(8.8)  $30,000  $30,001- 1(2.9)  $60,000  >$60,000 1(2.9)  Not Reported 1(2.9) | 13.8 years (SD= 2.4) | | |
| Rendina, et al. 2018 The critical role... | | | Black 17(34)  Latino 15(30)  White 10(20)  Multiracial/Other 8(16)  Total 50(100) | 38.7 (10.4) | | | | Not Reported | High School/GED or less 9(18)  Some College/Associate’s 28(56)  Bachelor’s/Other 8(16)  4-year degree  Graduate degree. 5(10) | | |
| Rendina, et al., 2018  Situational HIV stigma... | | | Black 18(33.9)  Latino 15(28.3)  White 11(20.8)  Multiracial/Other 8(15.1)  Not Reported. 1(1.9)  Total 53(100) | 38.6 (10.3) | | | | Not Reported | High School/GED or less 9(17)  Some College/ 29(54.7)  Associate’s degree  Bachelor’s/Other 9(17)  4-year degree  Graduate degree 5(9.4)  Not Reported 1(1.9) | | |
| Miner, et al., 2019 | | | Control  Caucasian 23(88.5)  African American 2(7.7)  Other 1(3.8)  Total Subsample. 26(100)  Hypersexual  Caucasian 8(61.5)  African American 1(7.7)  Hispanic 1(7.7)  Other 3(23.1)  Total Subsample 13(100)  Total 39(100) | 29.1 (7.9)  38.8 (10.7) | | | | Not Reported  Not Reported | Not Reported  Not Reported | | |
| Turner, et al., 2019 | | | Non-Starters  Black or African American 6(21.4)  Hispanic or Latinx. 11(39.3)  Other or multiple 6(21.4)  White 5(17.9)  Total Subsample 28(100)  Completed ≥ 4 of 7 EMAs  Black or African American 16(17.4)  Hispanic or Latinx. 27(29.35)  Other or multiple 22(23.9)  White 27(29.35)  Total Subsample 92(100)  Total 120(100) | 25.3(2.2)  28.4(5.9)  27.8(4.1) | | | | $0-  $250 9(32.1)  $251-  $600 6(21.4)  $601-  $1300 9(32.2)  ≥$1301 4(14.3)  Not Reported. 1(1.1)  $0-  $250 21(22.8)  $251-  $600 24(26.1)  $601-  $1300 21(22.8)  ≥$1301 25(27.2) | Less than Highschool 12(42.9)  High School/GED 5(17.9)  Some college or more 11(39.2)  Less than Highschool 8(8.7)  High School/GED 27(29.3)  Some college or more 57 (62.0) | | |
| Wray, et al., 2019 | | | White 59(73.8)  Black/African American 4(5.0)  American Indian/ 1(1.1)  Alaska Native  Asian 5(6.3)  Multiracial 6(7.5)  Not Reported 5(6.3)  Total 80(100) | 27.1(7.8) | | | | <$30,000 22(27.5) | College degree 41(51.3) | | |
| Carr, et al., 2020 | | White 76(76)  Black or African American 4(4)  Asian 8(8)  American Indian/Alaska Native. 1(1)  Multi-racial 6(6)  No Response. 5(5)  Total 100(100) | | | 27.1(7.7) | | <$30,000 29(29) | | | College Degree 54(54) | |
|  | |  | | |  | |  | | |  | |
|  | **Race/Ethnicity *n* (%)** | | | | **Age µ (SD**) | **Income *n* (%)** | | | | | **Education *n* (%)** |
|  | | | | | | | | | | | |
|  |  | | | |  |  | | | | |  |
|  | **Race/Ethnicity *n* (%)** | | | | **Age µ (SD**) | **Income *n* (%)** | | | | | **Education *n* (%)** |
| Wray & Monti, 2020 | White 76(76)  Black or African American. 4(4)  Asian 8(8)  American Indian/Alaska Native 1(1)  Multiracial 6(6)  No Response 5(5) | | | | 27.1(7.7) | <$30,000 29(29) | | | | | College Degree 54(54) |
| Wray, et al., 2020 | White 76(76)  Black or African American. 4(4)  Asian 8(8)  American Indian/Alaska Native 1(1)  Multiracial 6(6)  No Response 5(5) | | | | 27.1(7.7) | <$30,000 29(29) | | | | | College Degree 54(54) |
